# Properties of Packed Bed Structures Formed during Filtration: A Two and Three-Dimensional Model

**DOI:** 10.1021/acs.oprd.3c00147

**Published:** 2023-08-29

**Authors:** William Eales, Chris J. Price, William Hicks, Paul A. Mulheran

**Affiliations:** † Department of Chemical and Process Engineering, University of Strathclyde, Glasgow G1 1XJ, UK; ‡ CMAC, 99 George St, Glasgow G1 1RD, UK; § Chemical Development, Pharmaceutical Technology and Development, Operations, AstraZeneca, Macclesfield SK10 2NA, UK

**Keywords:** agglomeration, modeling, size distribution, packing fractions, percolation

## Abstract

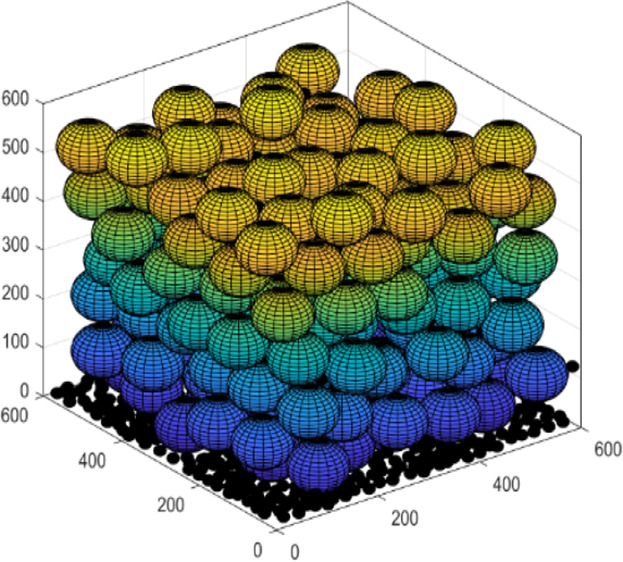

Agglomeration is an issue that causes many problems during secondary processing for pharmaceutical companies, causing material to need further processing and costing additional time and resources to ensure a satisfactory outcome. A potential source of agglomeration arises from the particle contacts established during filtration that lead to robust agglomerates forming during drying, so that a necessary first step toward understanding agglomeration is to study the packing properties of filtration beds. Here, we present two and three-dimensional models simulating the formation of packed bed structures during filtration. The models use circular and spherical particles of different sizes, mimicking the bimodal particle size distributions sometimes encountered in industrial practice. The statistics of packing and void formation, along with the distribution of interparticle contacts and percolation structures, are presented and discussed in the context of filtration, drying, and agglomeration. The model paves the way for predictive capabilities that can lead to the rational design of processes to minimize the impact of agglomeration.

## 1 Introduction

Of the many issues that can occur during secondary processing of Active Pharmaceutical Ingredients (APIs), agglomeration is one that can result in significant inconvenience for pharmaceutical companies.

**Figure 1:**
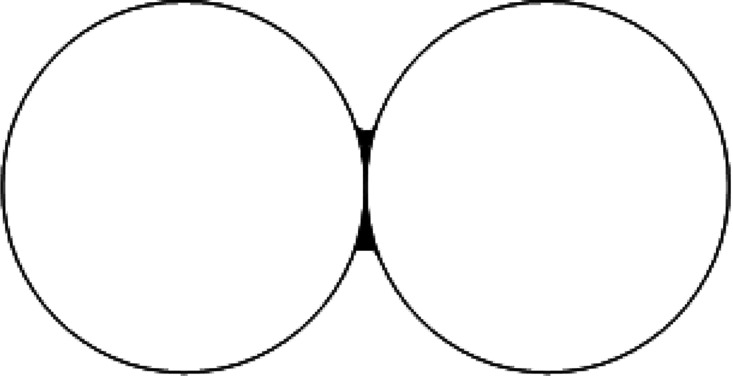
Schematic illustration of a bridge formed between two particles (redrawn from ref (1)).

Agglomeration can be defined as the process of single particles gathering into an agglomerate, which is a cluster of the individual particulate solids.^1^ This often occurs through the forming of bridges between particles during drying, as shown in Figure 1, when the solvent is evaporated leaving behind dissolved impurities and API deposited at the points of contact between the particles, holding them together.^2,3^

During drug processing, agglomeration can result in various issues. It can cause large variations in particle size distribution within the system, which negatively affects tableting as it becomes more difficult to ensure a consistent amount of API in each tablet.^4,5^ Maintaining content uniformity across the tablets is essential^6^ and multiple studies to determine the effectiveness of methods of ensuring content uniformity have been carried out.^7−13^ Impurities^14^ and mother liquor^15^ can also become trapped within agglomerates, which then require additional processing to fix, resulting in increased difficulty washing and drying agglomerates, and also contributing to difficulties during later stages of processing. Sufficiently hardened agglomerates can even damage the machinery used during secondary processing.^2^

While multiple studies have investigated the causes of agglomeration, little is still known about how to prevent it. If it was possible to determine how best to decrease, or fully eliminate, agglomeration within a system, it would be extremely useful as it would reduce the amount of additional processing needed to overcome the issues agglomeration causes. In order to develop strategies to reduce agglomeration, we first need to understand the relevant statistical properties of the filtration beds, such as how particles are interconnected and how this depends on the particle size distribution.

This paper discusses a two (2D) and three-dimensional (3D) model that has been created to study particle bed formation during the settling of particles under gravity of flow in filtration process, aiming for insight into bed characteristics and how they might affect secondary processing. Here, we model beds formed from circular (2D) and spherical (3D) particles for computational efficiency, noting that these choices align with experimental research being undertaken using glass spheres to investigate agglomerate properties.^16^ The models have been created in-house, instead of using pre-existing modeling tools such as EDEM,^17^ allowing bespoke analysis. This allows further investigation into agglomeration within the structures created and suggest potential avenues to combat it.

## 2 Methodology

The methodology is broken up into two sections, the initial section that creates the packed bed of particles and the secondary section that performs various analyses on the structures. FORTRAN^18^ has been used to code these models and MatLab^19^ was employed for visualization.

### 2.1 Development of Model 2D Packed Beds

#### 2.1.1 Setup

2D packed bed structures are built by the sequential addition of particles. These particles are assumed to be circular, with different radii permitted. The particles are constrained by a box whose dimensions are determined by the radii employed, so that the box length is 30× the largest particle radius used in the simulation.

Particles are first placed at the bottom of the box, choosing randomly from the desired particle radius distribution. The particles are positioned to avoid overlap, with the maximum separation ensuring that the smallest of the particles would not be able to fit in the ensuing gap. The box has hard walls against which the particles can rest, but they cannot overlap the walls. Once this process has been completed, the setup of the box is finished, and the box can then be filled through further sequential particle placements.

#### 2.1.2 Initial Placement of a Particle

**Figure 2:**
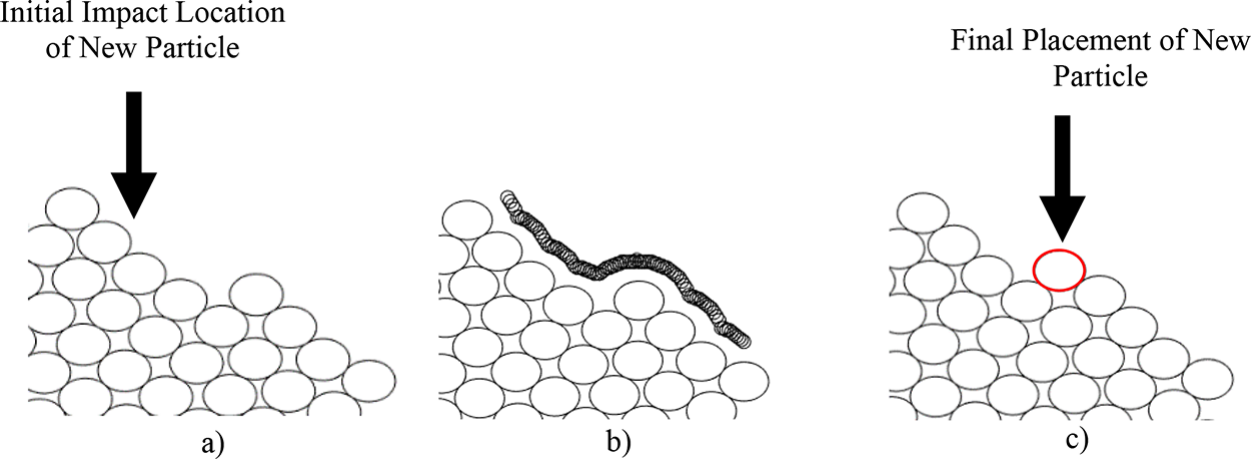
Stages the model completes when adding a new particle to the bed. (a, left): Bed prior to the addition of the new particle, showing the initial *x* coordinate chosen. (b, center): Possible sites for the new particle center that contact the existing bed. (c, right): Bed with the new particle having found its resting location.

Figure 2 shows the main stages the model goes through when placing a new particle on the bed. Initially, a random *x* coordinate for the particle center is determined, with the lower bound being the radius of the particle being entered, and the higher bound being the *x* coordinate of the righthand edge of the box minus the radius of the particle being entered. This is to ensure that the particle being added is entirely contained within the box and does not penetrate the walls.

The chosen *x* coordinate determines where on the pre-existing bed of particles the new particle will impact if it were to fall under gravity from above the bed, mimicking the gravitational settling of particles during filtration. An image of the area of impact is created using a grid with sites that overlap existing particles in the bed considered full, and the rest empty. The line of possible center points for the new addition is created so that it touches the top of the bed. Of course, many of these points will be unstable under gravity, so they must be searched systematically to identify the grid point where the particle will settle. Here, friction is neglected since we model particles only under gravity.

To perform the search, the impacted bed particle is first considered. If the center of the new particle is to the right of the center of the impacted particle, it polls through the aforementioned line of possible center points to the right, or vice versa if the particle impacts on the left of the impacted particle. The polling stops when the new particle would need to move to a point higher than the one it is currently on, signifying that it has reached a dip between two particles. This is therefore the stable site chosen since it is closest to the point of impingement having moved downhill under gravity.

#### 2.1.3 Refinement of Particle Position

**Figure 3:**
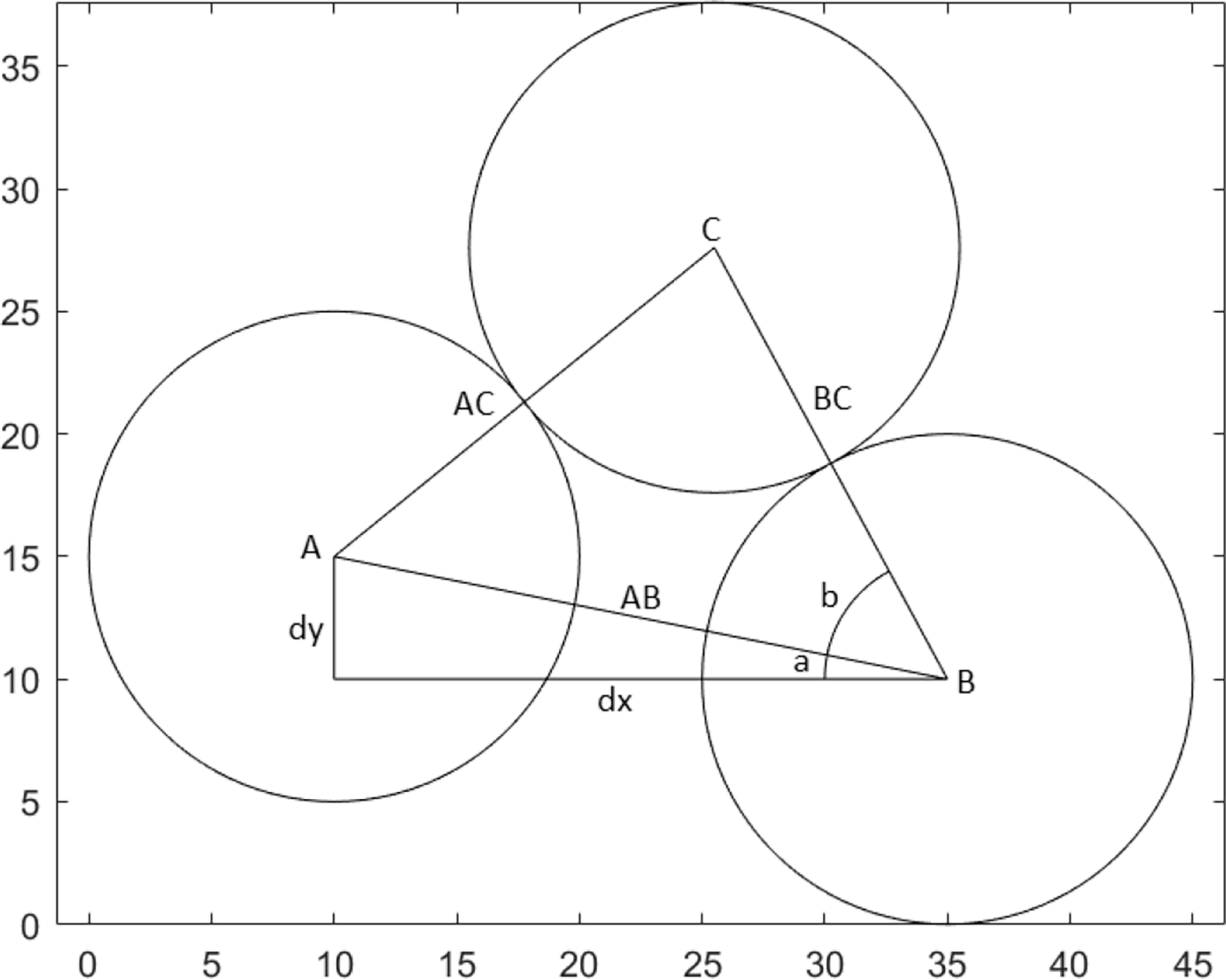
Angles and lengths used to calculate the final resting point of the new particle.

Up to this point, integer coordinates on a grid of possible sites have been employed to place the new addition. To identify the precise location for the resting place of the new particle, simple geometry is used as illustrated in Figure 3. Relevant angles between the lowest particle of the two pre-existing particles and the location of the new particle are determined using
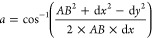
1
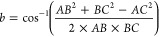
2where *AB* is the distance between particles A and B, etc., and (d*x*, d*y*) are the difference in (*x*, *y*) coordinates between particles A and B.

With the angles calculated, the gradients of the lines between the particle centers can be determined. Knowing that the distance is the sum of their two radii, the center point (*x*, *y*) of the new particle can then be determined.

The model then runs its final checks to ensure that no anomalous placements have occurred, i.e., ensuring that the new particle is not overlapping with any pre-existing particles, and that it is resting on two other particles. Once these final checks have been carried out, the new particle’s data is saved; the loop continues adding a new particle each time. When a failed particle placement occurs, either due to the added particle overlapping, being unbalanced, or being placed outside of the box, a counter is incremented. When the counter reaches a preset value, the box is considered full. At this point, the bed growth simulation ends with particle location, radii, and the contacts saved for the determination of statistical measures of the bed.

### 2.2 2D Bed Characteristics

#### 2.2.1 Number of Contacts between Particles

As well as saving the positions of each of the particles, the model also determines which particles are in contact which each other and saves this in a separate file. Particles are deemed in contact if the distance between them is equal to the sum of their radii. The second section of the model imports the packed bed data containing particle positions and contacts, to determine the void shapes and sizes that are present.

#### 2.2.2 Voids

**Figure 4:**
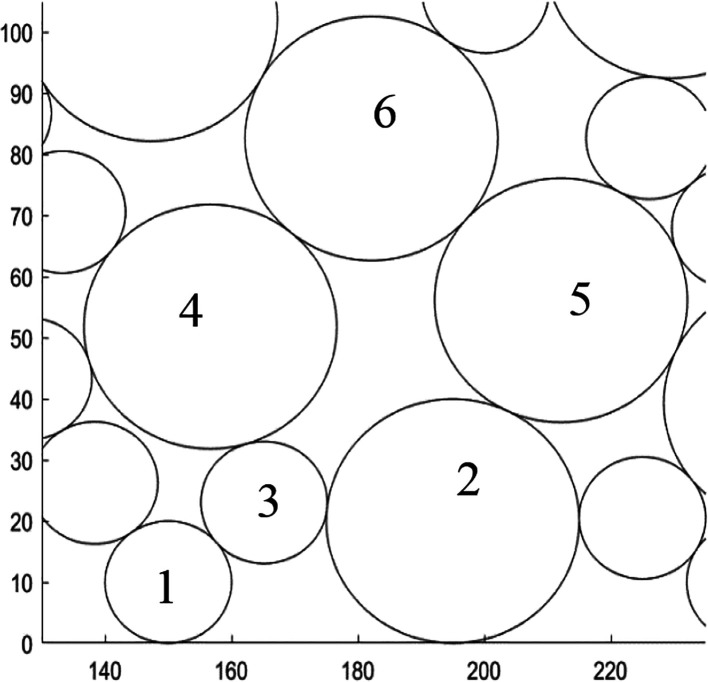
Order of particle addition yielding a void.

Each particle is processed in turn, searching recursively through the list of its contacts until a loop back to the original particle is found, as shown in Figure 4. Short loops are sought first, increasing the allowed loop size until all the relevant loops for the particle are found. The area enclosed by the loops is checked to ensure that they do not contain another particle center, and that all loops are unique.

For each valid loop, the area of the void inside is determined; this is done by first calculating the area of the polygonal shape defined by the centers of the particles, using
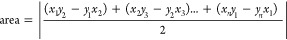
3where (*x_i_*, *y_i_*) are the coordinates of the *i*th particle in a loop containing *n* elements.

**Figure 5:**
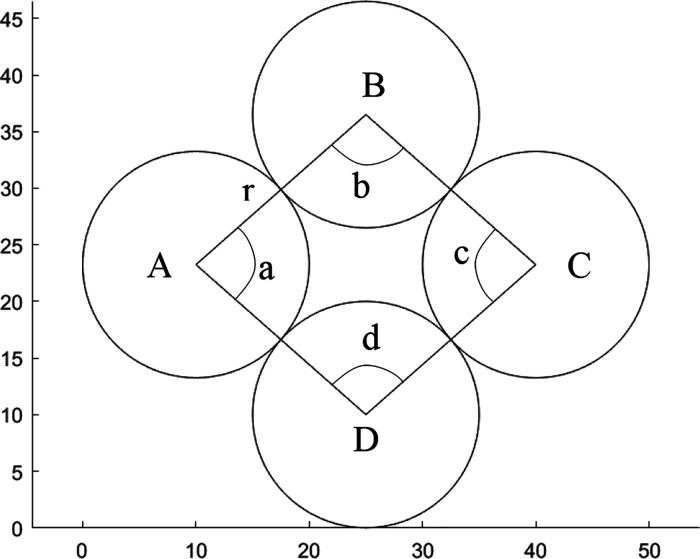
Angles and lengths used to calculate the area of individual voids.

The sector area of each of the particles on the inside of the polygon are calculated, using
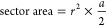
4where *r* is the radius of the particle and *a* is the angle subtended by the lines drawn to neighboring particle centers in the loop (see Figure 5).

The sector areas are then subtracted from the polygon area to find the void area.

#### 2.2.3 Percolation Structures

Determining the presence of percolation structures is done using the same method as searching for shapes in the grid; however, instead of looking to loop back to the first particle in the chain, it instead aims to reach the other side of the bed. Each particles’ contacts are ordered so that the model checks the particles closest to its destination first to reduce runtimes. Once a percolation chain has been found, its length and the particles that it is made up of are saved.

### 2.3 Modifications for 3D Beds

The three-dimensional model algorithm runs on many of the same principles as the two-dimensional version, with a few alterations, such as the introduction of the *z* axis into all aspects of the model, including the particle locations and the box size. Particles now must come to rest upon three particles instead of two. The three-dimensional algorithm does not use the equations discussed in Section 2.1.3, instead using a stochastic optimization method to precisely determine the new particle’s final resting place based on its distance from the particles it rests on. This algorithm works by taking the heatmap point closest to the resting point and making small random adjustments to the coordinates (in each dimension) until a position is reached that satisfies the previous 2D algorithm conditions, namely, resting on the correct number of particles in between their center points; and being the correct distance from the center of the particles and not overlapping with any other particle in the bed. These position adjustments start off large, scaling downward by a factor of 10 after every 500 adjustments. Once the distances between the position of the new particle and each of the particles it rests on equal (to within a tolerance of 10^–8^) the sum of the respective radii, the loop is exited, and the position saved. This method of placement calculation is effective and reliable.

### 2.4 Production Runs

The 2D and 3D models were used to produce 500 beds for each of the different bimodal size distributions investigated (see Table 1). The radii of the particles are in arbitrary units with respect to the grid used in the growth algorithm. The chance of placing a particle of either size was equal, so in the system with radius 10 and 20 particles (henceforth referred to as *r*_p_ = 10, 20), one *r*_p_ = 10 would be placed for every *r*_p_ = 20 on average. The packing fraction and particle contact numbers of each bed were then calculated.

**Table 1:** Packed Bed Systems Created with Different Particle Radii Present

system	radii present
1	10
2	10 & 20
3	10 & 50

In addition, 100 beds were created to determine individual void sizes with different radii of particles as shown in Table 1. 100 beds for the 2D beds and 200 beds for 3D beds were produced to gather percolation statistics.

## 3 Results and Discussion

### 3.1 2D Results

#### 3.1.1 Beds with Different Particle Radii Present

**Figure 6:**
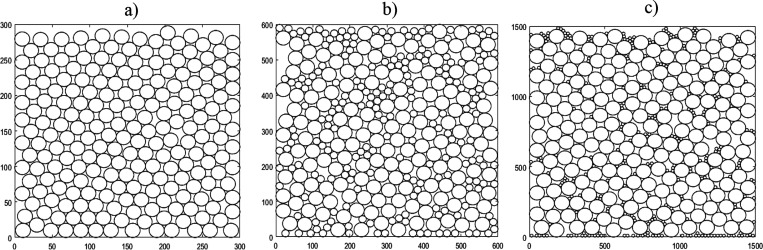
Packed beds of particles across three systems with different radii particles present. (a, left): *r*_p_ = 10. (b, middle): *r*_p_ = 10, 20. (c, right): *r*_p_ = 10, 50. Note that the size of the bounding box increases with the largest particle dimension.

The packed bed with only *r*_p_ = 10 present shows a mostly regular structure, as shown in Figure 6a, even with the irregularity of the initial placement of particles at the bottom of the bed. In the center of the bed, it can be seen that the average number for the contacts is four, since each addition contacts two existing particles, and a contact is shared by two particles. However, there are significant edge effects with the hard walls of the box.

The irregularity of the packing increases once the bed also includes larger *r*_p_ = 20, as shown in Figure 6b. As the *r*_p_ = 10 are not small enough to fit inside the voids created by the *r*_p_ = 20, they instead contribute to the increased irregularity in void shape and size as they force the larger, r_p_ = 20, to shift from a quasi-regular packing structure to accommodate for the smaller particles landing in between them.

This effect is still apparent in the bed containing *r*_p_ = 10, 50, however to a lesser extent, as shown in Figure 6c. Due to the larger difference in particle size, the smaller particles can fit in between the larger particles without greatly affecting the placement of the particles landing above them. There are still many instances of irregularity that spawn from the overabundance of smaller particles overfilling what might otherwise be a void, thereby forcing the addition of the next large particle to the side, preventing it from capping the putative void.

Note that the smaller particles filling in among the voids of the larger particles would increase the difficulty of washing the system, and the smaller particles forming clumps in between the larger particles would help bind them together, increasing the likelihood of agglomerates forming upon drying.

#### 3.1.2 Packing Fractions

The packing fraction of a system is calculated by first finding the total area of the system covered by particles, by summing the area of each particle within the system. This is then divided by the overall area of the system to find the fraction of the system covered by particle.

There is a slight correlation between the sizes of the particles present and the packing fraction. As shown in Table 2, the lowest packing average fraction occurs in the systems with only *r*_p_ = 10, as while it produces the most regular structures, this means that none of the voids between the particles are filled. In contrast, in the systems that contain larger particles, the smaller particles are able to sit in-between them, reducing the sizes of the void and therefore increasing the packing fraction, as shown by the *r*_p_ = 10, 20 and *r*_p_ = 10, 50 systems having higher packing fractions than the *r*_p_ = 10 systems. The higher average packing fraction in the *r*_p_ = 10, 50 systems, compared to the *r*_p_ = 10, 20 systems, is likely due to the order of the systems. As the *r*_p_ = 10, 50 systems are more regular due to the larger difference between particle size, it creates a more ordered system with the smaller particles filling in the voids. In contrast, in the *r*_p_ = 10, 20 systems, when the particles are of similar sizes, the smaller particles are not able to easily fill inside the gaps created by the larger particles, instead forming larger, more irregular voids and increasing the void fraction. When the particle radius ratio between smaller and larger particles is ≤0.4142:1,^20^ the smaller particles can rest inside the voids created by the larger particles.

**Table 2:** Packing Fractions from 500 Runs of the 2D Simulations with Different Particle Radii

	packing fraction		
particle radii	minimum	average	maximum
10	0.747	0.766 ± 0.007	0.806
10 & 20	0.757	0.779 ± 0.004	0.792
10 & 50	0.770	0.781 ± 0.005	0.796

Packing fractions have previously been investigated using random sequential adsorption (RSA) models, which calculate the maximum packing fraction to be roughly 0.547.^21^ Our values exceed this by about 0.2; while the packed beds presented here have a degree of randomness in the placement of the particles, the particles settle under gravity to create denser packing than with RSA, where particles are added at random without overlap, and no settling under gravity, until no more can be added.

The highest possible packing fraction for a bed of circular particles of the same size is ,^22^ so our values fall comfortably below this. The model will almost never achieve perfect packing in a triangular lattice due to the random nature of the bottom layer of the structure, unless the base layer is randomly placed perfectly next to each other.

#### 3.1.3 Number of Contacts between Particles

**Figure 7:**
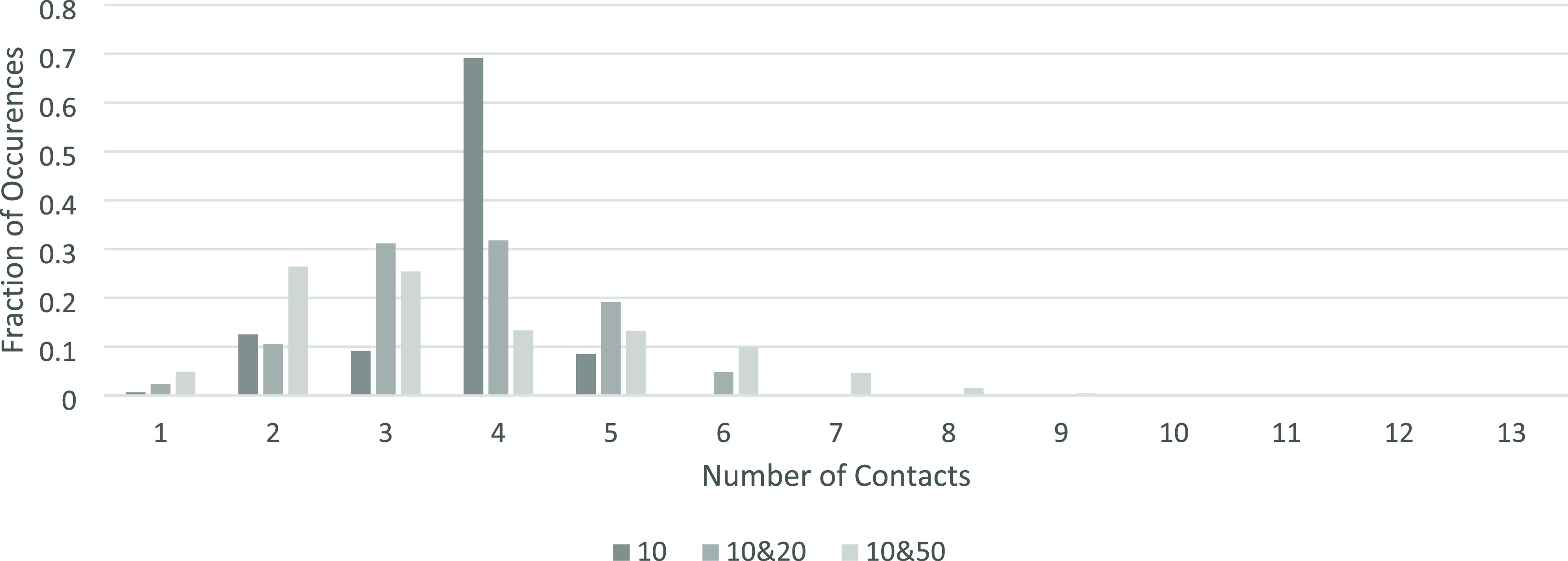
Frequency of the number of contacts each particle has across the 2D systems investigated.

The number of contacts each particle has was also investigated. As shown in Figure 7, across the different systems, particles will most often only have two to five contacts. Particles with fewer contacts than this are infrequent as having zero contacts requires being one of the initial particles placed on the bottom of the box with no particles lying on top, and one contact being a result of a particle resting against a wall and one other particle.

In the systems with only *r*_p_ = 10, there were very few particles with six contacts. In a perfectly ordered system, each of the particles would have six contacts as they would form a triangular lattice arrangement. As our systems have a degree of disorder within them due to the randomness of the particle placements, it is overwhelmingly unlikely for a full triangular lattice to occur randomly; however, it is possible for individual sections of the bed to form this structure. The majority of particles have four contacts, as expected, with two contacts both above and below.

**Figure 8:**
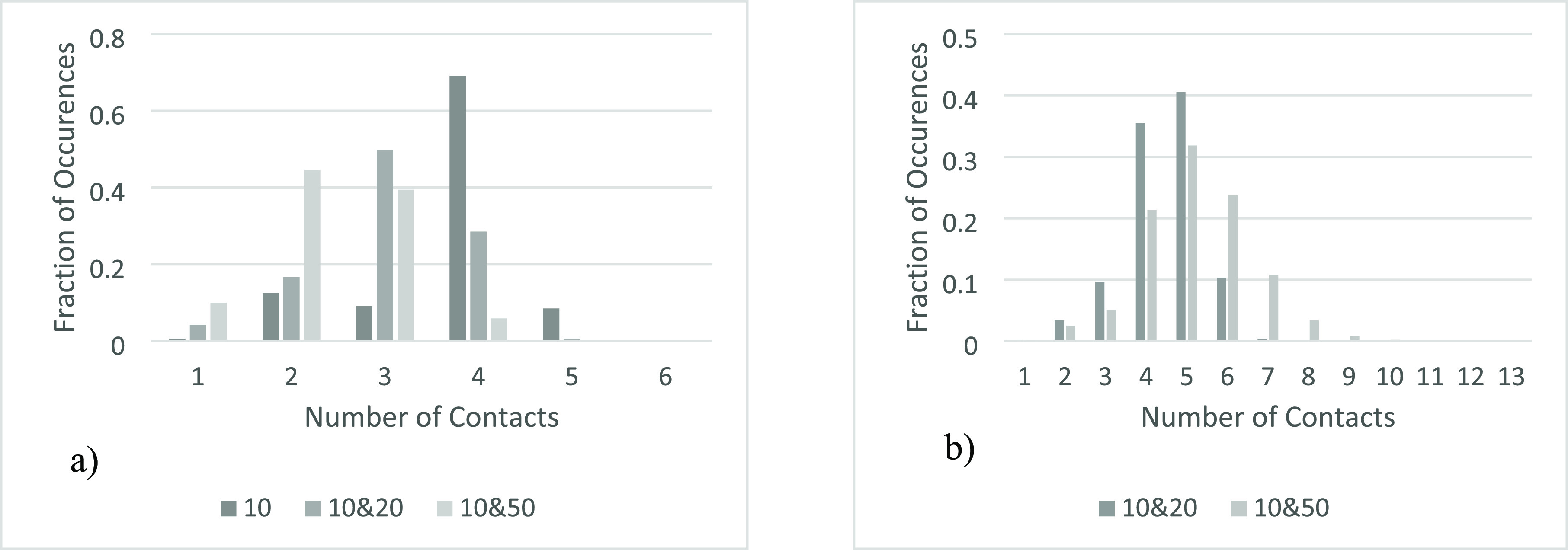
Frequency of the number of contacts each of the particles have across the investigated 2D systems separated by size. (a, left): *r*_p_ = 10. (b, right): *r*_p_ = 20 and r_p_ = 50.

As larger particles are added into the system with the *r*_p_ = 10, the number of contacts the particles can have increases, as the increased circumference of the larger particles allows for more contact points with smaller particles. In Figure 7, the highest number of contacts in the *r*_p_ = 10, 20 systems was seven, and in the *r*_p_ = 10, 50 systems it was thirteen. Figure 8a shows the frequency of each number of contacts for *r*_p_ = 10 across each of the systems created. Figure 8b shows the frequency of each number of contacts for larger particles, *r*_p_ = 20 and *r*_p_ = 50, present across each of the systems created.

When comparing the number of contacts of just the *r*_p_ = 10, there is a pattern across the different systems, with the majority of particles having 3 or 4 contacts. The main difference between systems is in the bed containing only *r*_p_ = 10; there is a large majority of particles with 4 contacts and very few with 3, compared to the other systems where the trend is reversed. This is because with only *r*_p_ = 10 in the bed, the rectangular structural motif, made up of groups of 5 particles with one in the center, is maintained throughout. In contrast, in systems with larger particles present, groups of solely smaller particles occur only in smaller clusters (see Figure 6).

The high frequency of particles with two contacts in the *r*_p_ = 10, 50 systems is due to small particles resting upon two others however not having any particles resting on them due to a larger particle capping the void above them.

#### 3.1.4 Individual Void Areas

The size of the individual void areas between particles in the system is another property of interest. Five repeats were done for each of the systems shown in Table 3 to get a good representation of the data.

**Table 3:** Smallest, Average, and Largest Void Areas in Three Simulations of the 2D Models Using Different Particle Radii Distributions

0	void areas			
particle radii	min.	average	max.	average void area scaled by largest particle area
10	16	78 ± 1.81	930	0.25
10 & 20	16	190 ± 5.72	3300	0.15
10 & 50	16	610 ± 23.34	10,000	0.08

As shown in Table 3, the size of the smallest void present does not vary across the different beds, which is due to the high likelihood of a triangle of *r*_p_ = 10 existing in all the beds formed, so that the smallest void will be the same size in all cases.

The average and largest void sizes increase as the width of the size distribution is increased, which is expected as there will be voids formed by just larger particles therefore having larger gaps in between them. To better compare these values, the average void sizes were scaled to be proportional to the size of the area of the largest particle present in system. Hence, for the *r*_p_ = 10 systems, its value was divided by 314.16, for the *r*_p_ = 10, 20 systems, its value was divided by 1256.64, and for the *r*_p_ = 10, 50 systems, its value was divided by 7853.98. As shown, these scaled sizes decrease with increased size distribution, as the voids between the particles are proportional to particle area; however, the larger particles allow the smaller particles to fit in between them filling up the gaps, whereas in the systems with more similarly sized particles, the gaps remain empty.

#### 3.1.5 2D Percolation Structures

**Figure 9:**
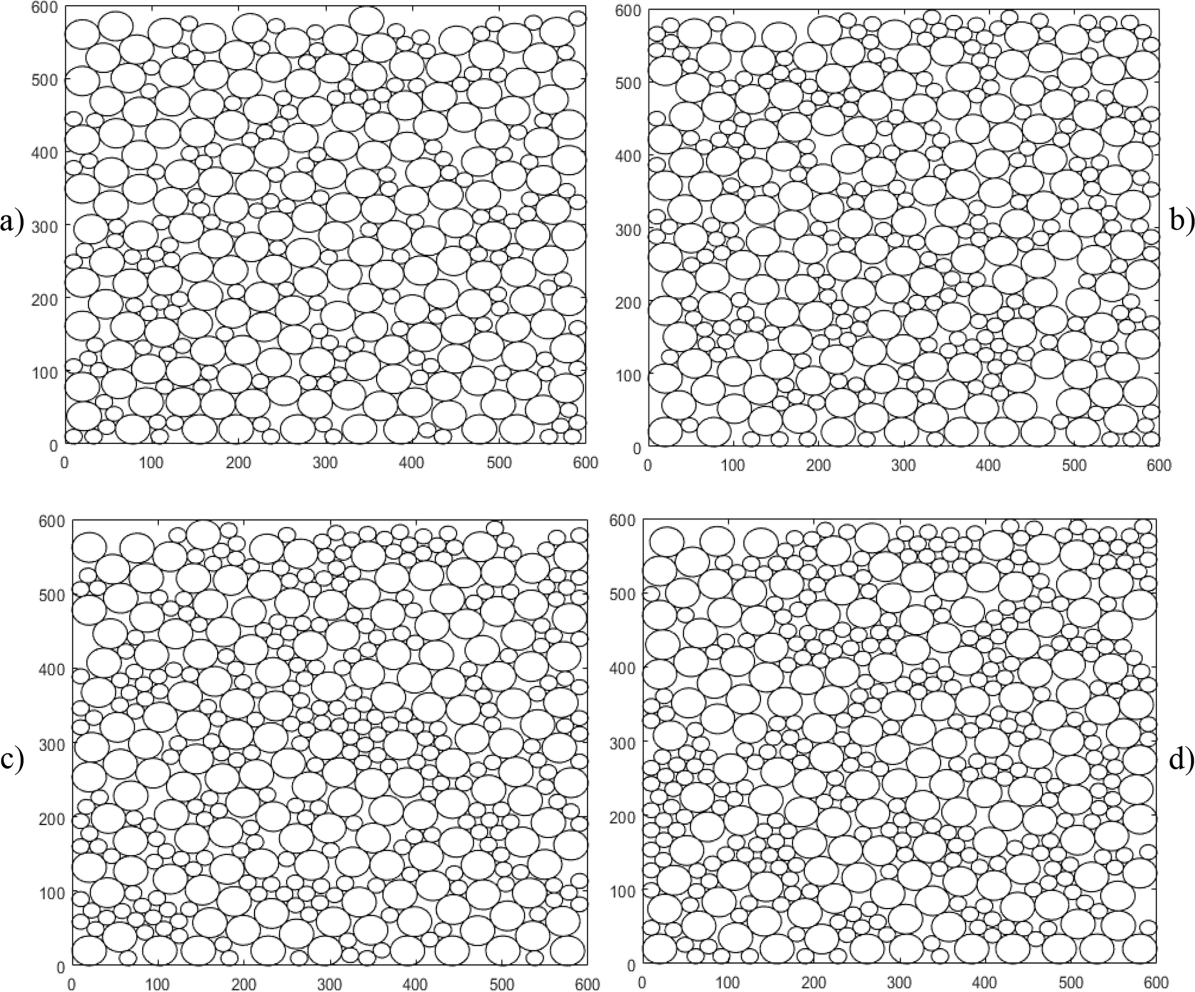
2D *r*_p_ = 10, 20 system with different proportions of large-to-small particles. (a, top left): 1:1, (b, top right): 1:1.5, (c, bottom left): 1:2, (d, bottom right): 1:2.5.

The existence of percolating structures^23,24^ in the packed beds is relevant to its structural properties. In Figure 9, structures formed with varying proportions of *r*_p_ = 20 to *r*_p_ = 10 are shown. Percolation pathways connecting large particles only from one side of the box to the other are also shown where they exist. Table 4 reports the results from 100 simulated beds to determine the percentage of systems that contained a percolating chain spanning the bed.

**Table 4:** Number of 2D Systems That Contained a Percolation Structure with Various Proportions of Large-to-Small Particles with Radii (*r*_p_) 10 & 20

particle proportion (large:small)	systems with percolation chains present (%)	minimum number of percolation chains present	average number of percolation chains present	maximum number of percolation chains present
1:1	98	0	102.73	400
1:1.5	82	0	113.19	319
1:2	59	0	62.29	300
1:2.25	33	0	27.27	350
1:2.5	23	0	17.48	200

As shown in Table 4, as the proportion (by number) of larger particles within the system decreases, it becomes more difficult for percolation chains to form; however, even at the lower ratios, there are still some chains present in our finite systems. The site percolation threshold for a regular triangular lattice is 0.523; however, due to the irregularity of our structures and the addition of smaller blocking particles, the threshold in these model bed structures could well be lower than this. The average and maximum numbers of percolation chains present also mainly decrease across the systems analyzed. The round number of the maximum chains data is due to there being a cap on the number of chains found per starting particle, so that the model does not run for too long.

Table 5 shows data concerning how many particles made up each percolation chain found, and, as expected, the length of chains shortens as the proportion of smaller particles increases, reflecting fewer available paths to take across the structure. The minimum lengths remain consistent for the same reason as the void sizes in Section 3.1.4; as the box has dimensions equal to 15 particles diameters, the minimum length of chain will be slightly above this value as it is unlikely to form a perfect straight chain across the bed.

**Table 5:** Length of Percolation Chains Found in 2D Systems with Different Ratios of Large:Small Particles

particle proportion (large:small)	minimum length of percolation chain present	average length of percolation chain present	maximum length of percolation chain present
1:1	18	66.28	108
1:1.5	16	41.98	81
1:2	17	32.10	66
1:2.25	17	32.69	62
1:2.5	18	30.04	59

### 3.2 3D Results

**Figure 10:**
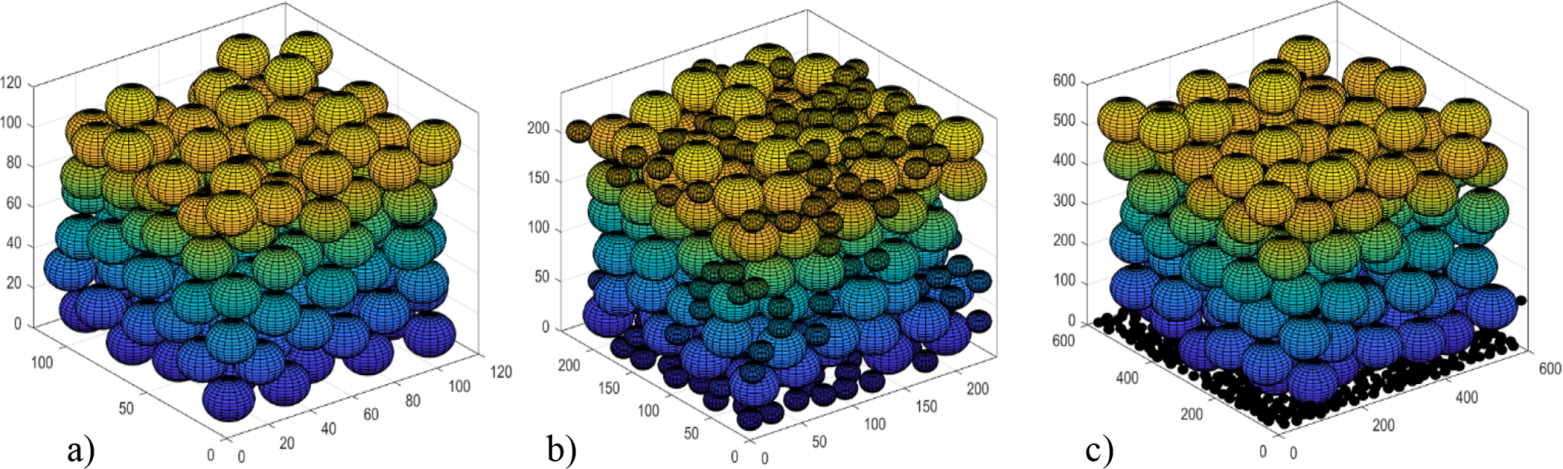
Packed beds of 3D particles across three systems with different radii particles present. (a, left): *r*_p_ = 10. (b, middle): *r*_p_ = 10, 20. (c, right): *r*_p_ = 10, 50. Note that the size of the bounding box increases with the largest particle dimension.

Figure 10 shows some examples of 3D packed beds, with different radii of particles present, that were created using the model presented in Section 2.3.

#### 3.2.1 Packing Fractions

The packing fractions determined for the 3D systems, using sum of particle volumes divided by box volume, follow a different pattern to that of the 2D systems. As shown in Table 6, the systems become more packed with the introduction of a larger sized particles, with the system that contained *r*_p_ = 10, 20 having a larger packing fraction than the other systems. As with the 2D systems, the smaller particles are able to fill in the voids between the larger particles when they are present; however, the 1:1 addition ratio of the particles sizes means that while the *r*_p_ = 10, 50 systems could have a higher packing fraction if the voids were filled with smaller particles, there are not enough small particles placed within the systems to fill the voids, thus leaving the *r*_p_ = 10, 20 systems with a higher packing fraction.

**Table 6:** Packing Fractions in 3D Packed Bed Systems with Different Particle Radii Present

	packing fraction		
particle radii	minimum	average	maximum
10	0.434	0.460 ± 0.007	0.475
10 & 20	0.480	0.497 ± 0.006	0.513
10 & 50	0.452	0.468 ± 0.006	0.486

**Figure 11:**
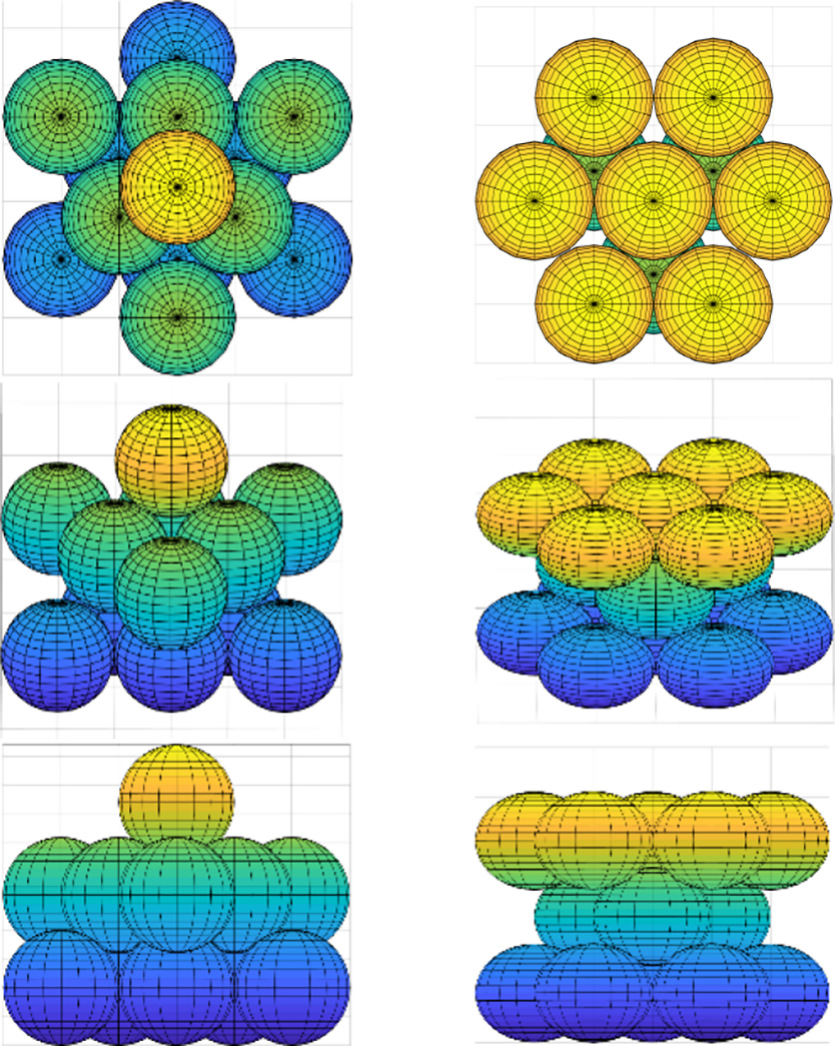
FCC lattice (left) and HCP lattice (right) (redrawn from ref (28)).

These values are lower than the highest packing fractions that have been calculated in systems of the same sized spheres. There are two lattices that can occur to achieve the highest packing fraction,^26^ which is (27) These two lattices, as seen in Figure 11, are face-centered cubic (FCC) and hexagonal close-packed (HCP).

Other examples of packing types and their maximum densities are the following: random close packing, 0.6400;^29^ the tetrahedral lattice, ;^30^ and the loosest possible density that has been found is 0.0555.^31^ Our values fit between these as expected as they are lower than the more packed systems due to our inherent randomness but more packed than the more irregular systems due to the presence of the simulated gravity forcing particles downwards to pack more tightly.

#### 3.2.2 Number of Contacts

In the FCC and HCP lattices discussed above, the expected number of contacts for each sphere is twelve, with three below, six on the same plane, and three above. However even the slightest irregularity causes the spheres on the same plane to be further away and no longer in contact with each other. Therefore, we are expecting our spheres to have six contacts on average, accounting for the three touching spheres above and below.

**Figure 12:**
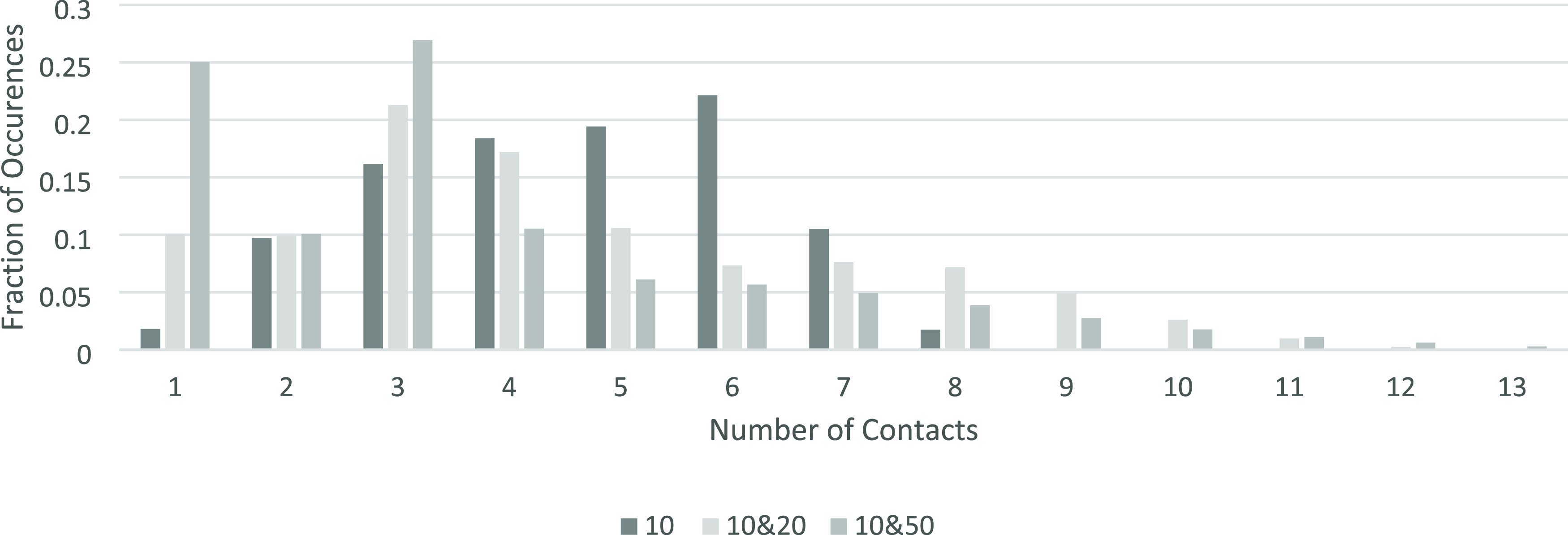
The frequency of the number of contacts each particle has across the investigated 3D systems.

As seen in Figure 12, the systems containing only *r*_p_ = 10 do show the most frequent contact number is six, however not by a large margin. Due to the large amount of disorder in these systems, the number of contacts ranges all the way from one to ten contacts in the single particle size bed.

The beds with different sized particles present peaks at three contacts, also with high occurrences of four to seven contacts. These are still around the expected value of six, with the lower ones being particles in contact with the edge of the box, due to the finite size effect.^25^

The lower end of the contact values is also due to particles that are in contact with the edges of the box as contacts between particle and boundary are not counted as well as smaller particles resting inside voids capped by larger particles. In the future, we are aiming to include those in the calculations to remove some of the finite size effect.

**Figure 13:**
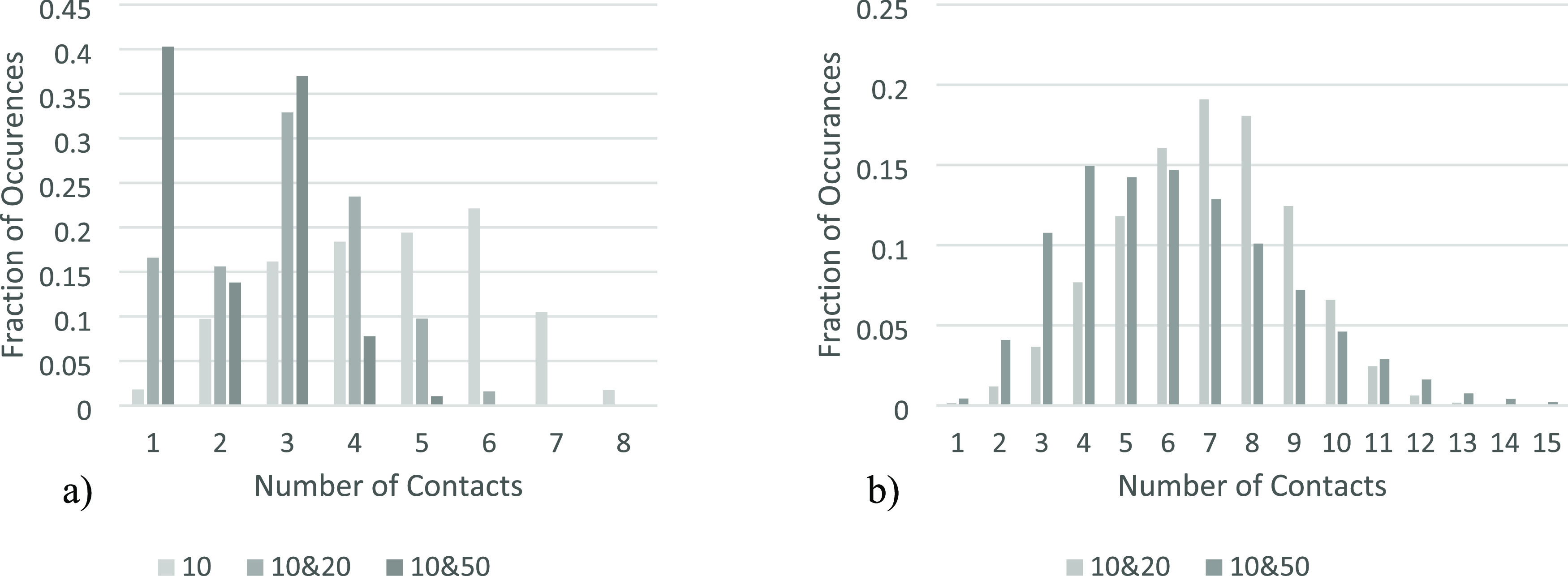
Frequency of the number of contacts each of the particles have across the investigated 3D systems separated by size. (a, left): *r*_p_ = 10. (b, right): *r*_p_ = 20 and r_p_ = 50.

As shown in Figure 13a, there is a large variation in the number of contacts the smaller particles have across the three investigated systems. The increased number of particles with one contact in the *r*_p_ = 10, 20 and *r*_p_ = 10, 50 systems is due to the higher box area and therefore more small particles falling to the bottom of the box, and only having a single contact with a particle resting above them. The large number of small particles with three contacts is due to a small particle resting on three larger particles with the void then capped above by another large particle, not allowing the smaller particle now trapped inside the void to gain any more contacts.

Figure 13b shows the difference between the number of contacts of the larger particles in the *r*_p_ = 10, 20 and *r*_p_ = 10, 50 systems. The *r*_p_ = 10, 50 data in Figure 13b is similar to the *r*_p_ = 10 data in Figure 13a as they are both forming a relatively ordered structure; however, the *r*_p_ = 10, 50 graph has a slower decline at the higher end of the number of contacts due to the smaller particles that will also be resting upon them.

#### 3.2.3 3D Percolation Structures

We also investigated the presence of percolation structures in the 3D systems. With the addition of the third dimension, chains spanning the box in either the *x* or *z* direction are sought using the same method as discussed in Section 2.2.3 for the 2D structures.

The 3D data shown in Table 7 show the same pattern as the 2D data in Table 4, with a higher frequency of percolation structures present when the number of large and small particle present are similar. However, there are still many more percolation structures present at higher ratios in the 3D systems compared to the 2D systems, with only 59% of systems containing a percolation structure in the 2D system with a ratio of 1:2 large:small particles, but the 3D system with the same ratio having 100% percolation presence. This is because in 3D, the particles tend to have more contacts, giving more options for the larger particles to connect to each other across the system. As shown, even at a proportion ratio of 1:6, the percolation threshold has not been found, and more percolation chains are being found than in the 2D system with a third of the ratio.

**Table 7:** Data on Percolation Structures in 3D Structures Containing *r*_p_ = 10, 20 Particles in Various Proportions

particle proportion (large:small)	systems with percolation chains present (%)	minimum number of percolation chains present	average number of percolation chains present	maximum number of percolation chains present
1:1	100	7	15.46	26
1:2	100	2	11.04	27
1:3	98	0	6.91	26
1:4	84	0	3.63	14
1:5	59	0	2.01	19
1:6	36	0	0.89	9

Again, as shown in Table 8, the minimum length of chain is close to the minimum possible but slightly above, with the 3D box dimensions being 6 particle diameters. The average chain lengths across the different ratios are consistent, likely due to the smaller size of the box resulting in much longer chains being unable to form. The maximum chain lengths are also relatively consistent compared to the 2D data, with a small decline still, again likely due to the comparatively smaller box size.

**Table 8:** Length of Percolation Chains Found in 3D Systems with Different Ratios of Large:Small Particles

particle proportion (large:small)	minimum length of percolation chain present	average length of percolation chain present	maximum length of percolation chain present
1:1	7	8.52	18
1:2	7	8.73	16
1:3	7	8.82	15
1:4	7	8.84	17
1:5	7	8.93	15
1:6	7	8.97	12

## 4 Summary and Conclusions

The models presented create a realistic representation of a packed bed of circular (2D) or spherical (3D) particles formed under gravity, with each particle coming to rest on the previously formed bed in a stable position. The model runs efficiently and can produce the particle bed systems rapidly, though there is a small decrease in speed the larger the particle array becomes.

The packing fractions in 2D systems created by the model were found to increase with the size distribution between the particle radii present within it, which is due to the smaller particles more easily able to fill the gaps between the larger particles. The 3D system packing systems were similar to this pattern, with an overall increase with broader size distributions; however, the trend was not linear in nature; the beds with *r*_p_ = 10, 20 were more densely packed than those with *r*_p_ = 10, 50.

When investigating the number of contacts for particles in the 2D beds, it was found that the majority of smaller particles had three or four contacts, as in the center of the bed each particle will be resting on two particles, and then have an additional particle or two resting on them. The larger particles had mainly five or six contacts; as with the smaller particles they would be resting on two others, however, with a larger circumference, they were able to have multiple extra smaller particles resting against them. The number of contact points in the 3D model follows a similar pattern, with the small particles having on average four to six contacts when on their own, but a spike of one and three contacts when in systems with larger particles. The larger particles have a higher average as their larger surface area allows more small particles to be in contact with them.

Percolation structures, comprising chains of large particles in contact, have been found within the beds produced both in 2D and 3D. The presence of percolating structures potentially impacts the way in which the beds will break under shear forces. The percolation threshold of a system could be a point at which the cohesiveness of a structure is changed due to the lack of percolation structures forming.

The ability to investigate these properties provides greater insight into the structure of the beds and the voids within them and will enable further research into secondary processing and agglomeration, allowing us to better determine what steps can be taken during filtration and drying to negate adverse effects, such as through how the beds form with varying size distributions or the effect percolation structures have on the stresses developed under shear at individual contact points. This model can also be used alongside experimental work, to compare and contrast the model’s outputs, and help us to control size distributions so that filter cakes can be processed more efficiently.

In future work, we will run further simulations to investigate how the ratio of large-to-small particles can affect the distribution of forces between the particles in the packed structures when they are stressed, exploring how cracks form through the bed. We are also aiming to expand the model’s capabilities to non-spherical particles so we can investigate other packed bed structures relevant to filtration, drying, and processing. On top of improving the model capabilities, experimental work is underway that can be used to verify the model outputs by investigating real systems and how particles pack within them.
